# Analysis of immediate 503 thyroid carcinoma deaths: trend of single institution in 2005–2024

**DOI:** 10.1530/ETJ-24-0368

**Published:** 2025-03-18

**Authors:** Haruhiko Yamazaki, Kiminori Sugino, Kosuke Inoue, Ryohei Katoh, Kenichi Matsuzu, Wataru Kitagawa, Mitsuji Nagahama, Aya Saito, Koichi Ito

**Affiliations:** ^1^ Department of Breast and Thyroid Surgery, Yokohama City University Medical Center, Yokohama, Kanagawa, Japan; ^2^ Department of Surgery, Ito Hospital, Tokyo, Japan; ^3^ Department of Social Epidemiology, Graduate School of Medicine and School of Public Health/Hakubi Center, Kyoto University, Kyoto, Japan; ^4^ Department of Pathology, Ito Hospital, Tokyo, Japan; ^5^ Department of Surgery, Yokohama City University School of Medicine, Yokohama, Kanagawa, Japan

**Keywords:** cause of death, thyroid carcinoma, tyrosine kinase inhibitor

## Abstract

**Background:**

This study aimed to investigate the changes in histological types and causes of death associated with thyroid carcinoma (TC) before and after the introduction of systemic drug therapy.

**Methods:**

The records of 503 deceased patients treated for TC and with death from TC between January 2005 and June 2024 were reviewed in this retrospective cohort study. Multivariate logistic regression was applied to assess whether the number of patients with anaplastic TC (ATC) at diagnosis and the number of local-related deaths changed before and after the introduction of lenvatinib (i.e. 2005–2014 vs 2015–2024).

**Results:**

Of the 503 patients, 157 (31%) had ATC, 253 (50%) had papillary TC (PTC), 67 (13%) had follicular TC (FTC), 17 (3%) had poorly differentiated TC, and nine (2%) had medullary TC. Respiratory insufficiency was the most common fatal condition, occurring in 192 cases (38%), followed by local-related death in 98 cases (19%) and brain-related conditions in 22 cases (4%). We found no difference in the frequency of patients with ATC at diagnosis (32 vs 30%; *P*-value = 0.772) and the frequency of local-related deaths (19 vs 20%; *P*-value = 0.736) between 2005–2014 and 2015–2024. These findings were supported by multivariate logistic regression models that adjusted for other covariates (adjusted *P*-value = 0.436 and 0.353, respectively).

**Conclusions:**

ATC, including anaplastic transformation from PTC and FTC, still accounts for approximately 40% of thyroid cancer deaths after the introduction of systemic drug therapy. Respiratory insufficiency is the most common immediate cause of death.

## Introduction

Thyroid carcinoma (TC) is the most common endocrine malignancy (1) and is mainly classified into five histological types: papillary TC (PTC), follicular TC (FTC), medullary TC (MTC), poorly differentiated TC (PDTC) and anaplastic TC (ATC), based on the World Health Organization (WHO) classification of thyroid tumors (2). Although the prognosis of PTC and FTC is generally good, some patients develop local or distant metastatic recurrence and have a poor prognosis (3, 4). MTC accounts for approximately 2% of all thyroid malignancies, and approximately 20–30% of all MTC cases are hereditary diseases (3, 5). PDTC is a rare tumor with a similar prognosis to DTC and ATC (6). ATC is one of the most lethal cancers among all human malignancies (7), accounting for only approximately 1% of all thyroid malignancies and approximately 40% of thyroid cancer deaths (3, 8).

In 1999, Kitamura *et al.* investigated the immediate causes of death in 161 patients with TC at Ito Hospital (8). The histological diagnoses of the patients in this study were ATC in 62 cases and DTC in 99 cases (PTC, *n* = 81; FTC, *n* = 18) at initial diagnosis, and 37 patients with DTC were diagnosed with anaplastic carcinoma at the time of death (8). Among 106 patients with specific causes of death, respiratory insufficiency was the most common fatal condition (*n* = 46, 43%), followed by local-related death, including hemorrhage from the tumor and airway obstruction (*n* = 30, 28%) (8). Therefore, from the viewpoint of histological type, improving the prognosis of ATC may be important for improving the prognosis of TC as a whole. Furthermore, from the viewpoint of the immediate cause of death, control of lung metastasis and local disease is considered important.

Since effective treatment options for metastatic or recurrent TC are limited, palliative surgery or external radiation therapy was performed for these patients. However, since 2014, various systemic agencies have admitted its use in clinical practice (9, 10, 11, 12, 13, 14, 15, 16, 17). In particular, lenvatinib has shown higher efficacy and has been shown to prolong the overall survival (OS) of patients with radioactive iodine-refractory DTC (18). On the other hand, lenvatinib is considered to have limited efficacy in ATC (19, 20). However, lenvatinib is effective in some patients with ATC and is still used in Japanese clinical practice (21, 22). Paclitaxel, a microtubule-stabilizing drug, also has some efficacy against ATC (12). Previously, we reported that patients with ATC who underwent curative resection after neoadjuvant paclitaxel therapy had a better prognosis, and curative resection significantly reduces the frequency of airway obstruction by local tumors as a cause of death (23).

Approximately 10 years have passed since systemic drug therapy has been fully introduced into Japanese clinical practice. However, there have been no detailed reports on the changes in histological types and causes of death in TC patients before and after the introduction of systemic drug therapy in Japan. This study aimed to investigate the changes in the histological types and causes of death associated with TC before and after the introduction of systemic drug therapy.

## Materials and methods

### Study participants

The study protocol was approved by the Ito Hospital ethics review board (approval no. 430) and performed in accordance with the 1964 Declaration of Helsinki and its later amendments. As this was a retrospective cohort study, the requirement for informed consent was waived.

Ito Hospital is now known for its high standard of care for thyroid diseases and handles 300,000 outpatient visits and 1,800 thyroid surgeries annually. We identified 671 deceased patients treated for thyroid cancer between January 2005 and June 2024. We retrospectively reviewed the electronic medical records of 671 patients and excluded 168 patients with insufficient medical records or deaths from other causes, such as other malignancies and unforeseen accidents. After all the exclusions, we included the remaining 503 patients in this study. After following up patients who were referred to another hospital, we confirmed the outcomes at the referral hospital by telephone inquiry or medical information form. Histological diagnoses were made according to the criteria of the Japanese Society of Thyroid Surgery (the latest edition of which was published in September 2023 (24)) based on the findings of aspiration biopsy cytology and pathological examination of surgical or biopsy specimens. The 8th edition of the AJCC/TNM staging system for thyroid cancer was used to evaluate clinicopathological factors (25).

### Statistical analysis

All statistical analyses were conducted using EZR (Saitama Medical Center, Jichi Medical University, Japan), which is a graphical user interface for R (The R Foundation for Statistical Computing, Austria) (26). Fisher’s exact test and chi-square test were used to compare categorical variables. The Mann–Whitney U test was used to compare continuous variables. Multivariate logistic regression models were applied to investigate independent factors for the frequency of patients with ATC, the cause of death, and anaplastic transformation among PTC patients. In the analysis for the frequency of ATC, we included sex, age at initial diagnosis, and the period of death (before the introduction of lenvatinib (2005–2014) vs after the introduction of lenvatinib (2015–2024)). In the analysis for the cause of death, we additionally included treatment strategy (surgery, external irradiation, chemotherapy and tyrosine kinase inhibitor (TKI)), distant metastasis site and anaplastic transformation (only among patients with PTC) in the model. In the analysis for anaplastic transformation among PTC patients, we included sex, age at initial diagnosis, treatment strategy (surgery, external irradiation, chemotherapy and TKI), distant metastasis site and the period of death. Given the small or no number of local-related deaths among patients with FTC, PDTC and MTC, we did not perform univariate and multivariate analyses for these histological types of TC.

## Results

### Baseline patient characteristics

The baseline characteristics of the patients are shown in Table 1. The histological types of the patients were as follows: ATC, *n* = 157 (31%); PTC, *n* = 253 (50%); FTC, *n* = 67 (13%); PDTC, *n* = 17 (3%); MTC, *n* = and 9 (2%). In our study, 214 patients were diagnosed with ATC at the time of death. Fifty-two and five patients were initially diagnosed with PTC and FTC, respectively. In these 57 patients, the duration between the initial diagnosis and anaplastic transformation was 9.7 years. Of the 503 patients, 146 (29%) were male and 357 (71%) were female. The median age at diagnosis was 64 years (range: 12–100 years), the median age at death was 73 years (range: 39–101 years), and the median survival from diagnosis to death was 85.2 months (range: 0.1–581.3 months), respectively. According to the TNM classification, 263 patients (52%) had T4 and 332 patients (66%) had N1. A total of 167 patients (33%) had distant metastases at the initial diagnosis.

**Table 1:** Baseline characteristics. Data are presented as *n* (%) or as mean (range).

Characteristics	Total	ATC	PTC	FTC	PDTC	MTC
*n*	503	157	253	67	17	9
Sex						
Male	146 (29%)	47 (30%)	66 (26%)	21 (31%)	7 (41%)	5 (56%)
Female	357 (71%)	110 (70%)	187 (74%)	46 (69%)	10 (59%)	4 (44%)
Age at initial diagnosis (years)	64 (12–100)	72 (36–100)	59 (12–91)	61 (34–87)	67 (35–87)	44 (34–69)
Age at death (years)	73 (39–101)	73 (39–101)	75 (39–95)	72 (45–90)	72 (43–87)	68 (47–87)
Survival from diagnosis to death (months)	85.2 (0.1–581.3)	5.3 (0.1–529.6)	151.3 (1.6–581.3)	128.0 (13.1–344.6)	66.5 (0.2–151.9)	174.7 (8.0–512.7)
TNM classification at diagnosis						
T						
T1	20 (4%)	0	16 (6%)	4 (6%)	0	0
T2	59 (12%)	2 (1%)	40 (16%)	13 (19%)	3 (18%)	1 (11%)
T3	92 (18%)	12 (8%)	38 (15%)	32 (48%)	7 (41%)	3 (33%)
T4	263 (52%)	143 (91%)	109 (43%)	3 (4%)	7 (41%)	1 (11%)
TX	69 (14%)	0	50 (20%)	15 (22%)	0	4 (44%)
N						
N0	60 (12%)	33 (21%)	10 (4%)	15 (22%)	2 (12%)	0
N1	332 (66%)	119 (76%)	191 (75%)	6 (9%)	8 (47%)	8 (89%)
NX	111 (22%)	5 (3%)	52 (21%)	46 (69%)	7 (41%)	1 (11%)
M						
M0	296 (59%)	71 (45%)	172 (68%)	38 (57%)	7 (41%)	8 (89%)
M1	167 (33%)	85 (54%)	46 (18%)	26 (39%)	9 (53%)	1 (11%)
MX	40 (8%)	1 (1%)	35 (14%)	3 (4%)	1 (6%)	0
Treatment						
Surgery	411 (82%)	84 (54%)	239 (94%)	65 (97%)	14 (82%)	9 (100%)
External irradiation	220 (44%)	73 (46%)	105 (42%)	30 (45%)	11 (65%)	1 (11%)
Chemotherapy	142 (28%)	107 (68%)	29 (11%)	2 (3%)	4 (24%)	0
TKI	129 (26%)	40 (25%)	52 (21%)	21 (31%)	8 (47%)	8 (89%)
Lenvatinib	118 (23%)	37 (24%)	49 (19%)	18 (27%)	8 (47%)	6 (67%)
Sorafenib	22 (4%)	2 (1%)	13 (5%)	5 (7%)	0	2 (22%)
Vandetanib	9 (2%)	0	0	1 (1%)	1 (6%)	7 (78%)
Other	4 (1%)	2 (1%)	1 (<1%)	1 (1%)	0	0
Distant metastasis site at the time of death						
Lung	419 (83%)	125 (80%)	216 (85%)	56 (84%)	16 (94%)	6 (67%)
Bone	186 (37%)	29 (18%)	89 (35%)	54 (81%)	9 (53%)	5 (56%)
Brain	73 (15%)	15 (10%)	47 (19%)	10 (15%)	1 (6%)	0
Liver	60 (12%)	18 (11%)	28 (11%)	5 (7%)	4 (24%)	5 (56%)
Adrenal gland	19 (4%)	10 (6%)	5 (2%)	3 (4%)		0
Kidney	10 (2%)	4 (3%)	4 (2%)	1 (1%)	1 (6%)	0
Cardia	3 (1%)	3 (2%)	0	0	0	0
Period of death						
2005–2014	234 (47%)	75 (48%)	122 (48%)	31 (46%)	5 (29%)	1 (11%)
2015–2024	269 (53%)	82 (52%)	131 (52%)	36 (54%)	12 (71%)	8 (89%)

ATC, anaplastic thyroid carcinoma; FTC, follicular thyroid carcinoma; MTC, medullary thyroid carcinoma; PDTC, poorly differentiated thyroid carcinoma; PTC, papillary thyroid carcinoma; TKI, tyrosine kinase inhibitor.

Surgery was performed in 411 patients (82%), external irradiation was administered in 220 patients (44%), and chemotherapy was performed in 142 patients (28%). However, only 84 patients (54%) with ATC underwent surgery. Since the approval of sorafenib in 2014, TKIs have been used in Japanese clinical practice. Lenvatinib (*n* = 118; 23%) was the most commonly used TKI.

All TKIs were introduced for treating recurrent or metastatic disease but not as neoadjuvant therapy at initial thyroid surgery. Among TC patients treated with TKIs, the median durations of OS in ATC, PTC, FTC, PDTC and MTC were 6.8, 140.2, 148.3, 75.7 and 169.4 months, respectively. Among TC patients who were not treated with TKIs, the median durations of OS in ATC, PTC, FTC, PDTC and MTC were 4.3, 151.4, 127.9, 15.4 and 512.7 months, respectively.

Regarding metastasis at the time of death, the lung (*n* = 419, 83%) was the most common site of metastasis, followed by the bone (*n* = 186, 37%), brain (*n* = 73, 15%), liver (*n* = 60, 12%), adrenal gland (*n* = 19, 4%), kidney (*n* = 10, 2%) and cardia (*n* = 3, 1%).

### Immediate causes of death

The causes of death are listed in Table 2. In some cases, it was not possible to determine a single specific immediate cause of death, that is, in patients in whom serious conditions developed simultaneously in multiple organs or in patients in whom general weakness progressed gradually without specific verified organ failure (cachexia). Specific immediate causes of death were not identified in 165 of the 503 (33%) patients. The remaining 338 patients were analyzed in detail for specific causes of death. Respiratory insufficiency was the most common fatal condition, occurring in 193 cases (38%), followed by local-related death in 98 cases (19%) and brain-related conditions in 22 cases (4%). The rate of local-related death in patients with ATC (*n* = 52, 33%) was significantly higher than that in patients with PTC (*n* = 39, 15%) (*P* < 0.001). Furthermore, the rate of local-related death in patients with PTC was significantly higher than that in patients with FTC (*n* = 4, 6%) (*P* < 0.001).

**Table 2:** Causes of death.

	Total (*n* = 503)	ATC (*n* = 157)	PTC (*n* = 253)	FTC (*n* = 67)	PDTC (*n* = 17)	MTC (*n* = 9)
Specific cause of death	338 (67%)	105 (67%)	164 (65%)	47 (70%)	14 (82%)	8 (89%)
Local tumor related	98 (19%)	52 (33%)	39 (15%)	4 (6%)	3 (18%)	0
Airway obstruction	59 (12%)	34 (22%)	21 (8%)	3 (4%)	1 (6%)	0
Bleeding from tumor or trachea	23 (5%)	13 (8%)	7 (3%)	1 (1%)	2 (12%)	0
Esophageal stenosis	7 (1%)	3 (2%)	4 (2%)	0	0	0
Others	9 (2%)	2 (1%)	7 (3%)	0	0	0
Respiratory insufficiency	192 (38%)	41 (26%)	99 (39%)	37 (55%)	9 (53%)	6 (67%)
Pulmonary metastasis	147 (29%)	34 (22%)	81 (32%)	24 (36%)	5 (29%)	3 (33%)
Pneumonia	14 (3%)	5 (3%)	4 (2%)	4 (6%)	0	1 (11%)
Carcinomatous lymphangiosis	14 (3%)	1 (1%)	7 (3%)	3 (4%)	2 (12%)	1 (11%)
Pleuritis	14 (3%)	0	7 (3%)	5 (7%)	1 (6%)	1 (11%)
Pulmonary hemorrhage	2 (<1%)	0	0	1 (1%)	1 (6%)	0
Pulmonary infarction	1 (<1%)	0	0	0	0	1 (11%)
Brain related	22 (4%)	5 (3%)	14 (6%)	3 (4%)	0	0
Brain metastasis	16 (3%)	4 (3%)	10 (4%)	2 (3%)	0	0
Cerebral infarction	2 (<1%)	0	2 (1%)	0	0	0
Cerebral hemorrhage	2 (<1%)	1 (1%)	1 (<1%)	0	0	0
Others	2 (<1%)	0	1 (<1%)	1 (1%)	0	0
Liver failure	5 (1%)	0	3 (1%)	0	0	2 (22%)
Renal failure	5 (1%)	0	3 (1%)	1 (1%)	1 (6%)	0
Disseminated intravascular coagulation	5 (1%)	3 (2%)	1 (<1%)	0	1 (6%)	0
Cardia related	4 (1%)	3 (2%)	1 (<1%)	0	0	0
Heart failure	3 (1%)	2 (1%)	0	0	0	0
Myocardial infarction	1 (<1%)	1 (1%)	0	0	0	0
Peritonitis	3 (1%)	1 (1%)	2 (1%)	0	0	0
Gastrointestinal bleeding	2 (<1%)	0	0	2 (3%)	0	0
Others	2 (<1%)	0	2 (1%)	0	0	0
Nonspecific cause of death	165 (33%)	52 (33%)	89 (35%)	20 (30%)	3 (18%)	1 (11%)

ATC, anaplastic thyroid carcinoma; FTC, follicular thyroid carcinoma; MTC, medullary thyroid carcinoma; PDTC, poorly differentiated thyroid carcinoma; PTC, papillary thyroid carcinoma.

#### Local-related death

Airway stenosis followed by bilateral vocal cord palsy or tracheal tumor invasion occurred in 59 patients. In 23 patients with tumor hemorrhage, uncontrollable bleeding arose from the tumor or the trachea. Seven patients had difficulty in oral intake due to esophageal stenosis caused by tumor invasion and died.

#### Respiratory insufficiency

Widespread multiple pulmonary metastases resulted in the replacement of a large amount of lung tissue by carcinoma in 147 of 192 patients who died of respiratory insufficiency. Lethal pneumonia, carcinomatous lymphangiosis and pleuritis were observed in 14 patients each. Pulmonary hemorrhage from lung metastasis or bronchi occurred in two patients. Pulmonary infarction occurred in one patient.

#### Brain-related causes

Cerebral herniation due to brain metastasis and cerebral infarction occurred in three and two cases, respectively. Cerebral hemorrhage from lung metastasis occurred in two cases. One patient died of meningeal carcinomatosis, and the other died of direct invasion by skull base metastasis.

#### Other causes

The other causes of death in 28 patients were as follows: liver failure, *n* = 5 cases; renal failure, *n* = 5; disseminated intravascular coagulation, *n* = 5; heart failure, *n* = 4; acute myocardial infarction, *n* = 1 case; peritonitis, *n* = 3 cases; gastrointestinal bleeding, *n* = 2; sepsis, *n* = 1 and superior vena cava syndrome caused by mediastinal lymph nodes, *n* = 1.

### Trends of histological type and causes of death

**Figure 1:**
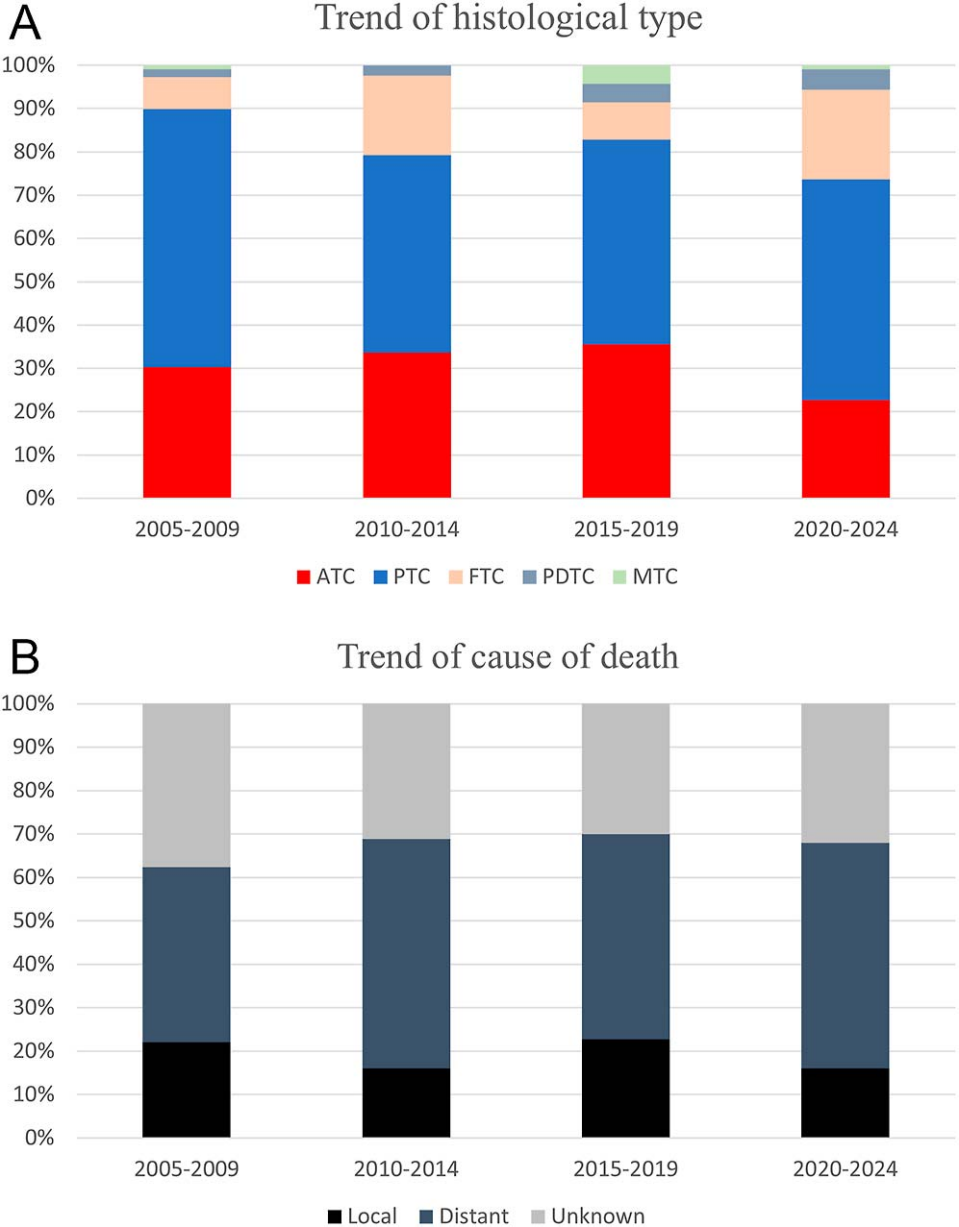
Trend of histological type and cause of death. (A) The numbers of papillary thyroid carcinoma (PTC), follicular thyroid carcinoma (FTC), medullary thyroid carcinoma (MTC), poorly differentiated thyroid carcinoma (PDTC), and anaplastic thyroid carcinoma (ATC) were 33 (30%), 65 (60%), 8 (7%), 2 (2%) and 1 (1%) in 2005–2009; 42 (34%), 57 (46%), 23 (18%), 3 (2%) and 0 in 2010–2014; 58 (36%), 77 (47%), 14 (9%), 7 (4%) and 7 (4%) in 2015–2019; and 24 (23%), 54 (51%), 22 (21%), 5 (5%) and 1 (1%) in 2020–2024. (B) The numbers of causes of death due to local-related factors, distant metastasis, and unknown reasons were 24 (22%), 44 (40%) and 41 (38%) in 2005–2009; 20 (16%), 66 (53%) and 39 (31%) in 2010–2014; 37 (23%), 77 (47%) and 49 (30%) in 2015–2019; and 17 (16%), 55 (52%) and 34 (32%) in 2020–2024.

The numbers of deaths in each 5-year period between 2005 and 2024 were as follows: 2005–2009, *n* = 109; 2010–2014, *n* = 125; 2015–2019, *n* = 163 and 2020–2024, *n* = 106. Trends in the histological type and cause of death are shown in Fig. 1A. In terms of histological type at the time of death, ATC accounted for 29–46% of cases. The number of patients with ATC at the diagnosis was 75 (32%) in 2005–2014 (before the introduction of lenvatinib) and 82 (30%) in 2015–2024 (after the introduction of lenvatinib), showing no significant difference between these periods of death (*P* = 0.772). There was also no significant difference in the frequency of patients with ATC at the time of death between 2005–2014 and 2015–2024 (105 (45%) vs 109 (41%); *P* = 0.366). We also found no relationship between the period of death and the frequency of ATC at the diagnosis (*P* = 0.436) and ATC at the time of death (*P* = 0.166) in the multivariate logistic regression model.

The trends in the causes of death are shown in Fig. 1B. Local-related deaths occurred in 98 patients (19%). The numbers of local-related deaths in each 5-year period between 2005 and 2024 were as follows: 2005–2009, *n* = 24; 2010–2014, *n* = 20; 2015–2019, *n* = 24 and 2020–2024, *n* = 17. In terms of the causes of death, local-related death accounted for 16–23%. The number of local-related deaths was 44 (19%) in 2005–2014 and 54 (20%) in 2015–2024, showing no significant difference between these periods of death (*P* = 0.736). We also found no relationship between the period of death and local-related death in the multivariate logistic regression model (*P* = 0.487; Table 3).

**Table 3:** Cause of death and clinicopathological factors. Data are presented as *n* (%) or as mean (range).

	Total (*n* = 503)				ATC (*n* = 157)				PTC (*n* = 253)				FTC (*n* = 67)		PDTC (*n* = 17)	
	Local (*n* = 98)	Non-local (*n* = 405)	*P*-UV	*P*-MV	Local (*n* = 52)	Non-local (*n* = 105)	*P*-UV	*P*-MV	Local (*n* = 39)	Non-local (*n* = 214)	*P*-UV	*P*-MV	Local (*n* = 4)	Non-local (*n* = 63)	Local (*n* = 3)	Non-local (*n* = 14)
Sex			0.62	0.913			1	0.903			0.435	0.615				
Male	26 (27%)	120 (30%)			16 (31%)	31 (30%)			8 (21%)	58 (27%)			1 (25%)	20 (32%)	1 (33%)	6 (43%)
Female	72 (73%)	285 (70%)			36 (69%)	74 (70%)			31 (79%)	156 (73%)			3 (75%)	43 (68%)	2 (67%)	8 (57%)
Age at initial diagnosis (years)	70 (40–94)	63 (12–100)	<0.001	0.036	74 (44–94)	72 (36–100)	0.997	0.610	63 (40–91)	59 (12–85)	0.006	0.021	71 (62–73)	59 (34–87)	59 (47–75)	68 (35–87)
Age at death (years)	76 (45–94)	73 (39–101)	0.081		74 (45–94)	72 (39–101)	0.862		77 (52–92)	74 (39–95)	0.027		77 (71–87)	70 (45–90)	59 (49–82)	72 (43–87)
Treatment																
Surgery	60 (61%)	351 (87%)	<0.001	0.001	20 (38%)	64 (61%)	0.011	0.059	33 (85%)	206 (96%)	0.011	0.149	4 (100%)	61 (97%)	3 (100%)	11 (79%)
External irradiation	40 (41%)	180 (44%)	0.571	0.743	17 (33%)	56 (53%)	0.018	0.116	19 (49%)	86 (40%)	0.378	0.159	2 (50%)	28 (44%)	2 (67%)	9 (64%)
Chemotherapy	40 (41%)	102 (25%)	0.716	0.231	34 (65%)	73 (70%)	0.716	0.525	6 (15%)	23 (11%)	0.414	0.714	0	2 (3%)	0	4 (29%)
TKI	19 (19%)	110 (27%)	0.123	0.203	15 (29%)	25 (24%)	0.561	0.689	3 (8%)	49 (23%)	0.031	0.258	0	21 (33%)	1 (33%)	7 (50%)
Distant metastasis site at the time of death																
Lung only	42 (42%)	122 (30%)	0.022	0.157	22 (42%)	42 (40%)	0.863	0.964	18 (46%)	65 (30%)	0.064	0.116	1 (25%)	7 (11%)	1 (33%)	6 (43%)
Period of death			0.736	0.487			0.397	0.598			0.729	0.865				
2005–2014	44 (45%)	190 (47%)			22 (42%)	53 (50%)			20 (51%)	102 (47%)			2 (50%)	29 (46%)	0	5 (36%)
2015–2024	54 (55%)	215 (53%)			30 (58%)	52 (50%)			19 (49%)	112 (53%)			2 (50%)	34 (54%)	3 (100%)	9 (64%)
Anaplastic transformation	-	-	-	-	-	-	-	-	17 (44%)	35 (16%)	<0.001	0.003	0	5 (8%)	-	-

ATC, anaplastic thyroid carcinoma; FTC, follicular thyroid carcinoma; PDTC, poorly differentiated thyroid carcinoma; PTC, papillary thyroid carcinoma; *P*-UV, univariate *P* values; *P*-MV, multivariate *P* values; TKI, tyrosine kinase inhibitor.

### Relationship between cause of death and clinicopathological factors

As no significant changes were observed in the trends of histological type and causes of death before and after 2014, further investigations were performed to determine whether there was a relationship between the cause of death and clinicopathological factors in each histological type (Table 3).

In the 157 patients with ATC at the initial diagnosis, there were no significant differences in sex, age at the initial diagnosis, age at death, rate of chemotherapy administration or TKI, sites of distant metastasis, and the period of death between ATC patients with local-related death and non-local-related death in the univariate analyses. ATC patients with local-related death had significantly lower rates of surgery (*P* = 0.011) and external irradiation (*P* = 0.018) compared to those with non-local-related death. However, these relationships were not statistically significant after adjusting for other variables in the multivariate analysis (surgery: *P* = 0.059; external irradiation: *P* = 0.116).

Of the 253 patients with PTC at the initial diagnosis, there were no significant differences in sex, rate of external irradiation, rate of chemotherapy administration, sites of distant metastasis, and the period of death between PTC patients with local-related death and non-local-related death in the univariate analysis. PTC patients with local-related death had significantly higher age at the initial diagnosis (*P* = 0.006) and age at death (*P* = 0.027) and had lower rates of surgery (*P* = 0.011) and TKI therapy (*P* = 0.031) than PTC patients with non-local-related death. Furthermore, anaplastic transformation occurred more frequently in PTC patients with local-related death than in those without (*P* < 0.001). Multivariate analysis also showed the relationship of age at the initial diagnosis (*P* = 0.021) and anaplastic transformation (*P* = 0.003) with local-related death.

Since there was a significant difference in the cause of death between PTC patients with and without anaplastic transformation, a further analysis was performed to determine whether there were differences in baseline characteristics in those patients. Table 4 presents the results. In the univariate analysis, there were significant differences in sex (*P* = 0.075), age at the initial diagnosis (*P* = 0.579), age at death (*P* = 0.23), rate of surgery (*P* = 0.495), sites of distant metastasis (*P* = 0.326) and the period of death (*P* = 0.437). However, PTC patients with anaplastic transformation were treated by external irradiation (*P* = 0.027), chemotherapy (*P* < 0.001) and TKI therapy (*P* = 0.033) more frequently than those with classic PTC. In the multivariate analysis, we found the relationship of age at the initial diagnosis (*P* = 0.049), external irradiation (*P* = 0.049) and chemotherapy (*P* < 0.001), with anaplastic transformation among PTC.

**Table 4:** Clinical characteristics of papillary thyroid carcinoma patients (*n* = 253) with classic and AT. Data are presented as *n* (%) or as mean (range).

	AT (*n* = 52)	Classic (*n* = 201)	*P*-UV	*P*-MV
Sex			0.075	0.203
Male	19 (37%)	47 (23%)		
Female	33 (63%)	154 (77%)		
Age at initial diagnosis (years)	61 (38–81)	59 (12–91)	0.579	0.049
Age at death (years)	73 (51–89)	75 (39–95)	0.23	
Treatment				
Surgery	48 (92%)	191 (95%)	0.495	0.866
External irradiation	29 (56%)	76 (38%)	0.027	0.049
Chemotherapy	20 (38%)	9 (4%)	<0.001	<0.001
TKI	5 (10%)	47 (23%)	0.033	0.149
Distant metastasis site at the time of death				
Lung only	20 (38%)	63 (31%)	0.326	0.115
Period of death			0.437	0.678
2005–2014	28 (54%)	94 (47%)		
2015–2024	24 (46%)	107 (53%)		

*P*-UV, univariate *P* values; *P*-MV, multivariate *P* values; TKI, tyrosine kinase inhibitor; AT, anaplastic transformation.

In the 67 patients with FTC and 17 patients with PDTC at the initial diagnosis, local-related deaths were observed in four patients (6%) and three patients (18%), respectively. Among nine patients with MTC, no one died by local-related death.

## Discussion

TC is the most common endocrine-related malignancy, with an incidence of 821,214 new cases diagnosed in 2022 (27). The incidence of thyroid cancer has risen substantially in the past 30 years in several high- and medium-income countries, although the rates of increase vary between and within populations (28, 29). According to Miranda-Filho *et al.* PTC was the main contributor to the overall incidence of thyroid cancer in all studied countries (30). In terms of thyroid cancer incidence, PTC accounts for most of all histological types, whereas ATC accounts for approximately only 1–2% in Japan (30). In contrast, 31% of patients in our study had ATC and 50% had PTC. Therefore, ATC accounted for a high proportion of deaths compared to its incidence. In 2022, Megwalu *et al.* reported thyroid cancer incidence and mortality trends in the United States in 2000–2018 and revealed that thyroid cancer-related mortality in 2018 increased in comparison to 2000 among all TCs (3). However, mortality increased in PTC and ATC but not in FTC or MTC (3). Therefore, improving the mortality rates of PTC and ATC appears to be a common challenge for both Japan and the United States.

In our study, anaplastic transformation occurred in 57 (18%) of 320 DTC patients. Because ATC is a lethal disease, this frequency might be noteworthy. To date, the mechanism of anaplastic transformation has not been fully understood. However, a recent study provided more reliable evidence that ATCs are derived from DTCs (31). *BRAF* and *RAS* mutations represent early oncogenic events during DTC development, and *TP53* and *TERT* promoter mutations probably occur as additional late events for ATC to develop from DTC (31). The median duration between the initial diagnosis and anaplastic transformation was 9.7 years in our study. If new treatments can prolong survival in DTC patients with local recurrence or distant metastasis, it will be necessary to pay more attention to anaplastic transformation.

In general, it has been reported that thyroid cancer patients are more likely to die from diseases other than thyroid cancer (32, 33, 34, 35). However, these studies used large databases and did not provide detailed information on the immediate causes of death from thyroid cancer. According to reports, distant metastasis is more often the immediate cause of death from thyroid cancer than local lesions (8, 36, 37). Park *et al.* reported the clinical course from diagnosis to death in 79 DTC patients who died between 1996 and 2018 (37). Among the 79 patients, respiratory failure (27.8%) was the most common cause of death, followed by airway obstruction (24.1%), complications due to immobilization (13.9%), brain metastasis (8.9%), cachexia (7.6%) and liver failure due to hepatic metastasis (2.5%) (37). In addition, none of the 17 patients with FTC died from airway obstruction, whereas it accounted for 30.6% of the deaths among 62 patients with PTC (37). Similarly, our study showed a significant difference in the rate of local-related death between patients with PTC and FTC. Our study included patients with TC who died between 2005 and 2024, the most recent period of data, and revealed that distant metastasis-related death, including respiratory failure due to lung metastasis, still accounts for the majority of immediate causes of death. In addition, it was also indicated that the cause of death may differ depending on histological type.

Morbidity and mortality due to TC can be caused by locoregional progression or distant metastases. As such, both locoregional and distant metastatic controls are important. The development of targeted therapies has shifted the landscape of advanced thyroid cancer management, and systemic therapies are usually introduced for treating recurrent and distant metastatic disease. In fact, previous studies have shown the possibility that some targeted therapies prolong OS in TC (18, 38). Although neoadjuvant treatment has emerged as a promising strategy to reduce surgical morbidity and allow inoperable tumors to become surgically resectable and resected with more optimal margins, the introduction of targeted therapy in the context of neoadjuvant therapy remains relatively nascent in advanced thyroid cancer. In 2020, Maniakas *et al.* evaluated OS in patients with ATC diagnosed between 2000 and 2019 (38). The study revealed that the patients undergoing surgery following neoadjuvant BRAF-directed therapy had a significantly favorable prognosis (38). Considering that BRAF-directed therapy was approved in Japan in November 2023, OS in Japanese patients with ATC is expected to improve. In addition, our findings are believed to be important for comparing the impact of BRAF-directed therapy on the frequency of different causes of death. Recently, the efficacy of targeted therapy used at neoadjuvant setting has also been reported not only in ATC but also in DTC and MTC (39, 40, 41, 42). These studies indicated that neoadjuvant therapy had the possibility to improve local tumor resectability. The study by Pitoia *et al.* that included 27 advanced thyroid cancer patients showed that DTC patients treated with lenvatinib achieved greater tumor reduction with more R0/R1 resection (40). In our study, lenvatinib was the most used TKI, and it was not used as a neoadjuvant setting. While we await the results of ongoing clinical trials, insight from the real-world application of lenvatinib using the neoadjuvant setting may inform future clinical trial development (41). A recent consensus statement published jointly by the International Thyroid Oncology Group and the American Head and Neck Society addressing mutational testing in thyroid cancer highlights the importance of assessing patients with advanced thyroid cancer for targeted treatment, including in the neoadjuvant setting (43). The use of targeted therapies, including lenvatinib, may change dramatically in Japan as well. The introduction of targeted therapy as a neoadjuvant setting may improve control of the local primary thyroid tumor, and it probably contribute to reduce the number of local-related deaths in advanced thyroid cancer patients.

The present study was associated with several limitations, including its single-center, retrospective design. In addition, it was not possible to determine a single specific immediate cause of death in some cases. However, autopsies are rarely performed in Japanese clinical practice unless death is clearly unnatural. Despite these limitations, this study provides important results. To our knowledge, this is the largest study to investigate the immediate causes of death in patients with thyroid cancer.

ATC, including anaplastic transformation from PTC and FTC, still accounts for approximately 40% of thyroid cancer deaths. Respiratory insufficiency is the most common immediate cause of death. In addition, multi-kinase inhibitors, including lenvatinib, seemed to have no impact on the immediate cause of death in thyroid cancer. Targeted therapy based on molecular testing is expected to become mainstream systemic therapy in the future. In addition, these systemic therapies using as neoadjuvant therapy setting are promising treatment strategy. Our findings are believed to be important for investigating trends in histological type and cause of death in thyroid cancer.

## Declaration of interest

The authors declare that there is no conflict of interest that could be perceived as prejudicing the impartiality of the work reported.

## Funding

No specific grant was received from any funding agency in the public, commercial or not-for-profit sector for the publication of this report.

## Ethics approval

The study protocol was approved by an independent ethics review board (approval no. 430) and performed in accordance with the 1964 Declaration of Helsinki and its later amendments.

## Informed consent statement

The ethics review board waived the requirement for informed consent due to the nature of the retrospective cohort study.
